# Does Social Complexity Drive Vocal Complexity? Insights from the Two African Elephant Species

**DOI:** 10.3390/ani11113071

**Published:** 2021-10-27

**Authors:** Daniela Hedwig, Joyce Poole, Petter Granli

**Affiliations:** 1 Elephant Listening Project, K. Lisa Yang Center for Conservation Bioacoustics, Cornell Lab of Ornithology, Cornell University, Ithaca, NY 14850, USA; 2 ElephantVoices, 3236 Sandefjord, Norway; jpoole@elephantvoices.org (J.P.); pgranli@elephantvoices.org (P.G.)

**Keywords:** syntax, formant modulation, vocal repertoire, gradation, acoustic structure

## Abstract

**Simple Summary:**

The social complexity hypothesis (SCH) for communication predicts that species with complex social systems exhibit complex communication systems. Testing the SHC in a broad range of species can contribute to a better understanding of human evolution because a co-evolutionary runaway process between social and vocal complexity may have shaped human language. Here we compare patterns of vocal complexity between the two species of African elephants: the savanna elephant exhibiting a complex social organization and the forest elephant exhibiting a simpler social organization. We review the existing literature and present novel insights into the vocal communication system of the elusive forest elephant, along with a first direct comparison with savanna elephants. Our findings suggest that the African elephants may contradict the SCH, as well as other factors potentially shaping patterns of vocal complexity across species. A better understanding of vocal complexity in the two species of African elephants will depend on continuing advancements in remote data collection technologies to overcome the challenges of observing forest elephants in their dense rainforest habitat, as well as the availability of comparable data quantifying both structural and contextual variability in the vocal production of both species of African elephants.

**Abstract:**

The social complexity hypothesis (SCH) for communication states that the range and frequency of social interactions drive the evolution of complex communication systems. Surprisingly, few studies have empirically tested the SHC for vocal communication systems. Filling this gap is important because a co-evolutionary runaway process between social and vocal complexity may have shaped the most intricate communication system, human language. We here propose the African elephant *Loxodonta spec*. as an excellent study system to investigate the relationships between social and vocal complexity. We review how the distinct differences in social complexity between the two species of African elephants, the forest elephant *L. cyclotis* and the savanna elephant *L. africana*, relate to repertoire size and structure, as well as complex communication skills in the two species, such as call combination or intentional formant modulation including the trunk. Our findings suggest that *Loxodonta* may contradict the SCH, as well as other factors put forth to explain patterns of vocal complexity across species. We propose that life history traits, a factor that has gained little attention as a driver of vocal complexity, and the extensive parental care associated with a uniquely low and slow reproductive rate, may have led to the emergence of pronounced vocal complexity in the forest elephant despite their less complex social system compared to the savanna elephant. Conclusions must be drawn cautiously, however. A better understanding of vocal complexity in the genus *Loxodonta* will depend on continuing advancements in remote data collection technologies to overcome the challenges of observing forest elephants in their dense rainforest habitat, as well as the availability of directly comparable data and methods, quantifying both structural and contextual variability in the production of rumbles and other vocalizations in both species of African elephants.

## 1. Introduction

The various forms of animal social organization constitute adaptive strategies that enable individuals to maximize their fitness in the face of ecological constraints imposed on their survival and reproduction [1]. Vocal communication is often a crucial component of social behavior, enhancing the benefits and mitigating the costs associated with a species’ social organization as it serves to coordinate the interactions and, as such, maintain the relationships between individuals cooperating and competing within and between social groups [2]. The *social complexity hypothesis* for communication states that complex social organization drives the evolution of complex communication systems [3]. The underlying notion is that animals living in highly variable and complex social environments (e.g., individuals interacting frequently and in diverse ways), need to convey a broader range of information to coordinate their social interactions. Such increased expressiveness can be achieved via various mechanisms, including an increase in vocal repertoire size and acoustic variation within and between call types, as well as the combination of call types into larger utterances. A vocal mediation of social relationships, in turn, may further facilitate the formation of complex social organizations [4], particularly when the animals’ ability to interact directly is limited [5], giving way to a co-evolutionary feedback mechanism ratcheting up social and vocal complexity. Even though already mentioned by Darwin [6], surprisingly few studies have empirically tested the social complexity hypothesis for vocal communication systems (e.g., [4,7,8,9]). Some of those have offered mixed results and leave many aspects of social and vocal complexity unexplored [10]. Filling this gap is particularly important because a co-evolutionary runaway process between social and vocal complexity may have been a crucial driving force shaping the most intricate communication system, human language [5], the evolutionary origin of which remains unresolved. The open-ended generativity of human language broadly arises from two underlying mechanisms of speech production: the intentional modification of the vocal tract to vary the energy distribution across frequencies (the so-called ‘formant structure’) to produce vowels [11], and the flexible combination of acoustic units into more complex utterances, generally referred to as syntax [12]. How the occurrence of analogs of syntax and formant modulation relates to variation in social complexity across non-human species remains little understood. 

In the absence of a fossil record, comparative studies of the vocal behavior of closely related species showing distinct differences in social complexity are a powerful tool to better understand the evolution of complex communication skills, including key features of human language. Fission-fusion societies in which groups regularly split into smaller subgroups are excellent systems to pursue such studies. Such plasticity in social cohesion may create unique challenges to the maintenance of social relationships; as such, distinctive selective pressures act on underlying communicative abilities [13], for instance in order to keep contact, cooperate and re-enforce social bonds or dominance hierarchies. Human language may have evolved to facilitate the maintenance of long-term social relationships when frequent direct interactions became impossible in the fission-fusion societies of our hunter-gatherer ancestors [14,15]. Previous studies related to language evolution focused largely on primate vocal communication, which was long considered fundamentally different from human language. With a strict division between language and primate vocal communication increasingly dissolving [16], our understanding of the evolutionary origins of human language will benefit from broadening investigations to evolutionarily more distant taxa, but with similar sociality. 

**Figure 1:**
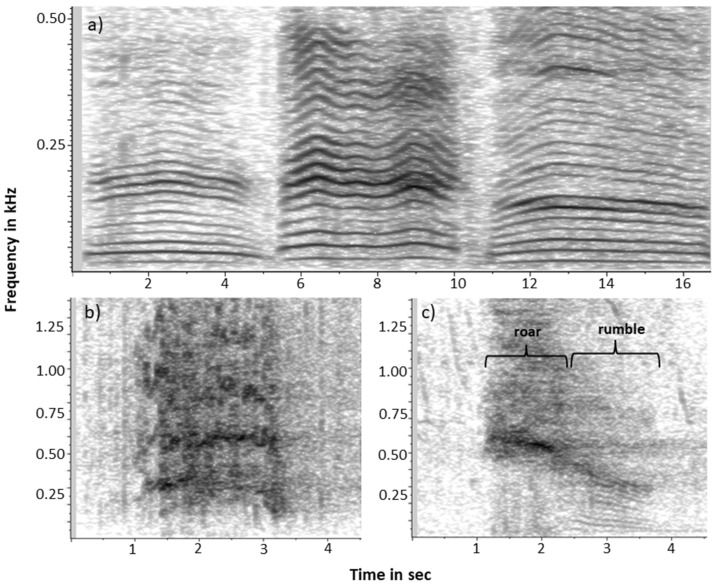
Representative examples of spectrograms of characteristic vocalizations of African forest elephants: (**a**) rumbles, (**b**) roar and (**c**) call combination consisting of a roar followed by a rumble.

We here aim to highlight the African elephant (*Loxodonta spec.*) as a highly suitable system in which to explore evolutionary pathways leading to complex communication skills. The two species, the forest elephant (*L. cyclotis*) and the savanna elephant (*L. africana*)*,* exhibit marked differences in the level of complexity in their respective fission-fusion societies. How these observed differences in social complexity relate to the two species’ vocal behavior remains poorly understood due to difficulties studying forest elephants in their dense forest habitat. A climatic transition to more arid conditions resulting in the reduction of forests [17] likely drove the divergence of savanna elephants from a forest elephant-like animal that preferred forest habitats between 2.6 and 5.6 million years ago [18,19,20]. While extant savanna elephants may inhabit a variety of habitats, including deserts, savannas, subtropical and temperate lowland and montane forests, forest elephants almost exclusively inhabit the dense tropical rainforests of Central Africa. Adult females of the primarily herbivorous savanna elephants exhibit complex multi-tiered fission-fusion societies, in which family units, consisting of a matriarch, her offspring and her close adult female relatives and their offspring, as well as more extended family “bond groups” may split and rejoin on a regular basis [21,22,23] while maintaining long-term social relationships within extensive social networks [24]. It is assumed that savanna elephants exhibit such strong social bonds because they benefit from cooperative defense against large pack-hunting predators (including humans), increased opportunities for social learning (allomothering, leadership) and sharing of knowledge about resource distribution [25,26,27,28]. In contrast, females of the more frugivorous forest elephants are found in smaller groups consisting of only one adult female with her dependent offspring likely due to elevated competition for food and the absence of non-human predators [29,30,31]. While multiple family groups exhibit stable and differentiated associations with one another, similar to the fission-fusion sociality of savanna elephants [32,33,34,35], the social networks of forest elephants appear smaller and lack multi-tiered structuring [36]. In both species, the coordination of social interactions within and between groups relies on characteristic rumble vocalizations. Rumbles are tonal calls with a rich harmonic structure and a low fundamental frequency that extends into the infrasonic range [37,38]. Both species habitually combine rumbles with broadband call types (laryngeal roars, cries, barks, here broadly referred to as roars, and sometimes with trumpets and snorts) into more complex combinatorial call types (Figure 1). 

In the following, we present the current state of knowledge on forest elephant vocal communication in comparison to savanna elephants, with direct reference to evidence for the social complexity hypothesis from other species. We present findings based on already published studies, as well as results based on new data used to conduct a first direct comparison between savanna and forest elephant vocal behavior. We discuss if our current assessment of differences in the vocal communication system supports the social complexity hypothesis and conclude with suggestions for future research.

## 2. Methods

To collect information on patterns of vocal complexity in the two species of African elephants we followed two approaches. First, we undertook a detailed literature review. These findings are indicated with the appropriate citation. Second, we collected new data on forest elephants, and re-analyzed already available data for savanna elephants, as described in the following. Results based on these new data are indicated under new findings in separate paragraphs.

### 2.1. Forest Elephants

Data collection on call contexts took place at Dzanga Bai, a forest clearing in Dzanga-Ndoki National Park in the southwestern Central African Republic (2.963° N, 16.365° E). The clearing is approximately 10 hectares in size, characterized by a sandy pan intersected by a permanent stream. Elephants enter the clearing for several reasons, primary among them being access to mineral rich water in small monopolizable depressions or pits that the elephants dig themselves [31].

We conducted behavioral observations on the contexts of calls produced mainly by unidentified individuals from September 2018 to April 2019 from an 8 m high observation platform at the edge of the clearing. We used opportunistic sampling paired with simultaneous audio-recording using an Earthworks omnidirectional microphone, capable of accurately recording the very low frequency calls of elephants, attached to a Sound Devices MixPre3 Audio recorder, and a 48 kHz sampling rate. For each audible call for which we were able to identify the caller and context with a high level of confidence, detailed notes on the behavioral contexts were taken. These notes were examined and collated into distinct context categories with the goal to compile the first ethogram of call contexts in forest elephants. Spectrograms of sound recordings were generated in Raven Pro Sound Analysis Software^®^ (version 1.5) using a Hann window with a frequency and time resolution of 0.98 Hz and 0.0255 s. Based on visual inspection of these spectrograms, we categorized calls into rumbles (N = 304), roar only (N = 88) and combination types: rumble then roar (RU-RO; N = 25), roar then rumble (RO-RU; N = 67), rumble, roar, rumble (RU-RO-RU; N = 38) and roar, rumble, roar (RO-RU-RO; N = 2). To ascertain that these call types can be assigned reliably, DH and a second observer independently scored a subsample of N = 75 calls. The percent agreement between the observers was 97%.

A subset of rumbles recorded with sufficient quality (high signal-to-noise ratio, no concurrent environmental noises) was used to investigate contextual differences in the structure of rumbles based on acoustic measurements conducted following the protocols described in Hedwig et al. [39]. We used discriminant function analysis and linear regression analysis to investigate whether rumbles can be assigned to the context categories based on their acoustic structure, focusing on the most frequently observed contexts and excluding the rare calls we recorded from adult males and infant calves (N = 246; see Table S1 in Supplementary Material for detailed sample sizes by context and age-sex class).

### 2.2. Savanna Elephants

We recorded elephants primarily in Amboseli, Kenya between 1986 and 1990, 1998 and 2006, and in 2020, from a population of known-aged individuals studied since 1972. To obtain suitable sample sizes of calls from individuals, we focused on one family of 27 elephants in 1999 and 2000. In 1998 we also recorded elephants in Laikipia and Maasai Mara, Kenya, and from semi-captive orphan elephants in Tsavo, Kenya. The vast majority of data included in this paper are from individually known callers from Amboseli. Through 1990 we used a Nagra IVSJ recorder; between 1998 and 2003 an HHB PDR 1000 DAT recorder and after 2003 a factory modified Nagra Ares BB. Most recordings were made with an Earthworks QTC1 omni-directional microphone. All our equipment was capable of accurately recording the very low frequency calls of elephants (down to at least 10 Hz; see Poole 2011 for more detail).

We made observations from a vehicle (except the Tsavo orphans). When a suitable group was found we parked near an individual or sub-group that provided good visibility (5–20 m to the nearest elephant). Once the elephants moved greater than about 25 m away, we moved the vehicle again. Although many elephant calls persist over long distances, the best quality calls and field data are typically from the closest individuals; accordingly, we varied the nearest elephant. We noted the specific location, group size and type and the individuals present. When we heard a call, we recorded the call type, call context-type, caller, distance to the source and any contextual or other comments about the situation or the behavior of the calling individual. We use the term *call type* to refer to the broad, structurally differentiated categories of sounds (e.g., rumble, roar, trumpet, etc.), and the term *context type* to refer to a priori subtypes initially differentiated from a combination of sound quality and social context (e.g., begging-rumble, musth-rumble, let’s-go-rumble, greeting-rumble). Many of these have since also been structurally differentiated [40]. We noted caller, call type and context-type with a level of confidence (A: certain, B: fairly confident, C: educated guess, D: no idea). A call context-type assigned confidence “A” required that both the behavioral context and sound quality matched the context-type designation. Elephant calls were systematically logged from field notes into a custom-designed FileMaker Database (N = 6642). 

Our long term studies of savanna elephants resulted in the recent release of The Elephant Ethogram [41], a detailed library of the communication and behavior of African savanna elephants, which provides a foundation for comparative studies focused on the vocal communication systems of the two species of African elephants. Building on from Poole and Granli [42], The Elephant Ethogram suggests and defines 23 behavioral contexts (https://www.elephantvoices.org/elephant-ethogram/ethogram-table.html, accessed on 23 October 2021), at least 20 of which are associated with vocalizations. To conduct a first direct comparison of the contextual use of rumbles between the two species of elephants, we have aligned the savanna elephant behavioral contexts (N = 15; and, within these, the specific call sub-types) that we believe best match the broader contexts currently described for forest elephants. Future studies will need to harmonize the contexts for a more accurate comparison.

To compare the behavioral contexts of rumbles, roars and combinations between the two species, we selected all SE rumbles that had been placed in behavioral contexts and had been assigned a context-type with an accuracy of A or B (N = 3006), and likewise, all SE roars and rumble-roar combinations with a context-type accuracy of A or B (roar only: N = 90; RU-RO: N = 12; RO-RU: N = 33; RU-RO-RU: N = 22). Pardo et al. [43] demonstrated that these call types can be assigned reliably with a mean percentage of agreement between observers of 90%. 

For a subset of rumbles, we used Signal 4.0 to take acoustic measurements. Due to the high frequency of overlapping calls, measurement was not automated, rather it was taken in the spectrograph view using the cursor. We used linear regression analysis to investigate contextual differences in the duration of rumbles, for which acoustic measurements and information on the age of the caller were available (N = 1177 rumbles recorded from 53 known individuals (median number of calls per individual = 5 [interquartile range 1–32]); see Table S2 in Supplementary Material for detailed sample sizes by context and age-sex class). 

## 3. Results

### 3.1. Rumble Repertoire Structure, Repertoire Size and Contextual Use

Social and vocal complexity can be described through diverse attributes [44]. Among the most used attributes of vocal complexity is the size of a species’ vocal repertoire. Across avian and mammalian taxa, vocal repertoire sizes relate to various aspects of social complexity, including group size [4,7,8,45,46,47], social and mating system [9,48,49,50], level of gregariousness [48], time spent grooming [4] and the number of social roles in a group [7]. However, exceptions in some taxa of frogs, birds and primates highlight that factors other than social complexity, or at least other aspects of it, may also shape patterns of vocal complexity across species [51,52]. Even though assumed to be a driving force behind the evolution of human language, how the degree of complexity of the hierarchical structure characteristic for fission-fusion societies relates to patterns of vocal complexity has not been investigated in a comparative perspective. Freeberg et al. [3], however, suggest that communicative requirements may be affected by the fact that relationships across the different tiers of the social system may have different qualities. Moreover, an aspect of vocal complexity even more overlooked is the level of structural variation within and between call types. Within-species comparisons of acoustic variation demonstrate that call types involved in coordinating often socially more complex close-range interactions exhibit particularly high variation compared to those used for long-distance communication [53,54,55,56], suggesting social complexity also plays a role in shaping the degree of gradation within vocal repertoires. 

The cornerstone of any research aiming to utilize the genus *Loxodonta* to elucidate relationships between social and vocal complexity is detailed quantitative information on the acoustic structure of their vocal repertoire, as well as its contextual use. Classification schemes and estimates of the size of the rumble repertoire of savanna elephants vary widely and remain unclear. Studies were either carried out in captive settings on a small number of individuals with a limited range of social contexts or on wild elephants, yet, often with small sample sizes per individual. Defining repertoire sizes is an inherently difficult endeavor often influenced by bias due to human perception or classification approaches (i.e., based on context with subsequent quantification of the acoustic structure of resulting context types versus based solely on quantifying acoustic structure), as well as the statistical tools used. Based solely on the acoustic structure, savanna elephant rumbles can be categorized into three to six highly intergraded types [57,58]. Others argue that savanna elephant rumbles constitute a single call type with graded variation [59]. Rumble repertoire assessments that also take into account the contextual use and vocal production mechanism of rumbles have proposed up to 19 rumble types [40,60,61]. Addressing the problem of small sample sizes in wild elephants, one study focused on a family of 27 individuals [40] and, using step-wise discriminant function analyses, classified 14 of these apparent rumble types given in specific contexts well above that expected by chance (57–79%) (54–81% with cross validation) [40]. These results suggest that, despite the highly graded acoustic structure of savanna elephant rumbles, there appear to be rumble sub-types given within specific narrow contexts. Behavioral responses to playback experiments (e.g., [62]) add further support to this assertion.

A quantitative assessment of the acoustic structure of forest elephant rumbles recorded at four sites across Central Africa using passive acoustic monitoring suggests that, compared to savanna elephants, the rumbles of forest elephants may exhibit a higher, but less modulated fundamental frequency, which reaches its maximum frequency earlier in the call and also exhibits higher formants [39]. Based solely on the acoustic structure, the results also indicate a possibly larger, but similarly highly graded, repertoire of rumbles. Using cluster analysis, forest elephant rumbles can best be classified into five to eight rumble types. While these rumble types were structurally highly graded, discriminant function analysis indicated they are predictable with a classification accuracy between 75% and 81% [39]. Interestingly, forest elephants used a larger set of seven rumble types when in a forest clearing compared to only four when in the forest, with four types observed exclusively in the clearing and one exclusively in the forest [39]. As passive acoustic recordings are entirely blind to the context in which recorded calls occurred, it remains to be ascertained how these categories reflect differences in age and sex of the callers or behavioral context.

### 3.2. New Findings

**Figure 2:**
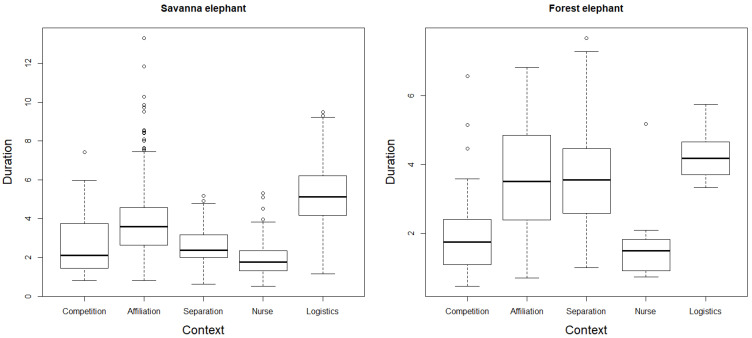
Relationship between context and the duration of rumbles produced by forest elephants (N = 246 calls) and savanna elephants (N = 1177). Statistical analysis for forest elephants excluded the contexts of sexual and anti-predatory, as well as adult males and infants due to the small sample size (see Table 1). These were included in the analysis for savanna elephants, but the two contexts were omitted in the figure for comparability. See Tables S3 and S4 in Supplementary Material for detailed results of the linear regression investigating how the duration of rumbles differed between contexts in the two species.

Our new data on the contextual use of rumbles by forest elephants collected at Dzanga Bai indicate that forest elephants produce rumbles in seven broad distinct contextual categories when in the clearing (Table 1). Similar to savanna elephants, forest elephant rumbles can, to some degree, be assigned to these different contexts based on their acoustic structure. Discriminant function analysis assigned 56% of forest elephant calls to the correct context, with call duration explaining most variation between contexts. Linear regression analysis, controlling for a positive correlation between caller age and call duration, indicated context had a significant effect on rumble duration (likelihood ratio test; x^2^ = 84.39, df = 4, *p* < 0.001, N = 246 forest elephant rumbles). While rumbles given in a logistical context were on average the longest, they were not significantly longer than those produced during affiliation and separation. Rumbles produced by calves during nursing were on average the shortest rumbles, with no significant difference from rumbles given during competition, but both competition and nursing rumbles exhibited significantly shorter durations than those produced during affiliation, separation and logistics contexts (Figure 2, see Table S3 in Supplementary Material for detailed model output).

**Figure 3:**
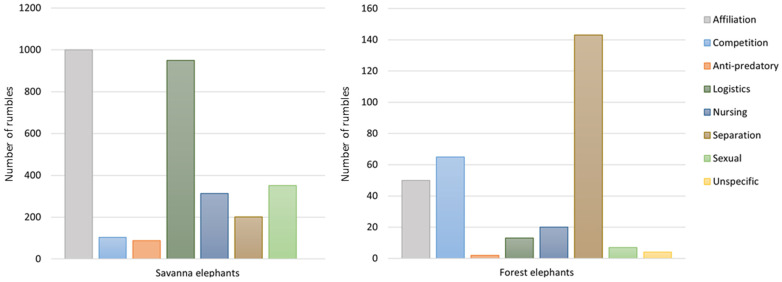
Number of rumbles produced by forest elephants at Dzanga Bai and savanna elephants in seven and eight broad context categories, respectively (savanna elephants: N = 3006, forest elephants: N = 304).

Our analysis suggests that both species use rumbles in similar broad contexts, but at different rates. Savanna elephants in Amboseli appear to produce a higher proportion of rumbles in affiliative, logistics and sexual contexts, in contrast to the high proportion of rumbles emitted in separation and competition contexts by forest elephants at Dzanga Bai (Figure 3). Part of this discrepancy though may be due to the categorization of contact-rumbles under logistical context in savanna elephants, while calls given in a similar context may have been categorized under a separation or an unspecific context in forest elephants. Exactly equivalent events and calling patterns involving contact-rumbling in savanna elephants have not been identified in forest elephants. In savanna elephants, contact-rumbles are unlike the separated-rumbles made by calves who are lost and looking for their mothers. Since the separation context defined for forest elephants is largely composed of calls by calves who are separated from their mothers, we chose to compare these with separated-rumbles and retain the contact-rumbles in the logistics context for the savanna elephants. 

Rumbles used by both species in the same broad contexts appear to be structurally similar. In both forest and savannah elephants, rumbles associated with nursing were significantly shorter than other rumbles, while those associated with logistics were longer than those produced in other contexts (Figure 2, see Table S4 in Supplementary Material for model output).

### 3.3. Formant Modulation

Mammalian vocal production follows principles similar to the production of human speech, conceptualized in the source-filter theory which decomposes the acoustic structure of vocalizations according to their mode of production [63,64]. A vocalization’s fundamental frequency reflects the frequency of the vibrations of the vocal folds (i.e., the source). As the sound subsequently travels through the vocal tract (i.e., the filter), the length and shape of the tract defines how energy is distributed, resulting in characteristic patterns of frequencies with particularly high energy, the so-called resonant frequencies or formants of the vocalization. Across mammals, including humans, body size correlates with fundamental frequency, formant frequencies and formant spacing [65,66,67,68,69], and fundamental frequency and its modulation vary with the motivational state of the caller, formalized by Morton (1977) as motivation-structural rules [70]. A hallmark of human speech is the intentional modification of the vocal tract independently of source-related components to produce vowels with distinct formant structure, enabling speakers to encode information about external entities independent of motivational state and body size [11,71]. Non-human vocal tracts were long considered uniform tubes allowing little variation in formant structure [72]. While more recent studies suggest that the vocal tract of monkeys can produce human-like vowels [16,73,74,75], and that variation in formant structure in monkey vocalizations can encode information about external contexts [76,77,78], it remains poorly understood if non-human mammals alter formant structure independently of fundamental frequency and motivational state. For instance, in Diana monkeys, formant structure varies independently of the fundamental frequency [78,79], whereas in baboons variation in formant structure is linked to fundamental frequency [75]. Moreover, it has been argued that even if correlated with fundamental frequency, formant modulation may not be a consequence of the motivational state alone [16]. A comprehensive simultaneous investigation of variation in formant structure and fundamental frequency in relation to body size, motivational state and the behavioral context in species exhibiting unusual flexibility in the size and shape of their vocal tract, such as elephants, is particularly suitable to further elucidate formant modulation in non-human mammals and the evolutionary origins of the mechanisms underlying speech production in humans.

Elephants exhibit unique morphological adaptations of the hyoid apparatus and pharynx, which allows for exceptional flexibility in the shape of their unusually elongated and plastic vocal tract in form of the trunk [80,81]. Such flexibility does not only enable savanna elephants to produce idiosyncratic, novel and imitated sounds [40,82,83] but it may be critical to the formation of the species’ complex social organization. Savanna elephants critically rely on their low-frequency rumble vocalizations to coordinate interactions and stay in contact with other family groups over large distances [37,40,84]. Savanna elephants may intentionally facilitate such long-distance vocal communication by actively including the trunk in vocal production thereby lowering the formant structure of rumbles, which likely renders them less prone to attenuation during sound propagation [60]. However, the functions of formant modulation in savanna elephants are not completely understood, as the energy distribution of rumbles also varies with other contextual factors (as can be seen in [40]) including information about nearby threats [85]. Passive acoustic monitoring recordings used to quantify the acoustic structure of forest elephant rumbles indicate that similar to savanna elephants [60,84,85,86], forest elephant rumbles also exhibit two formants below 250 Hz [39]. In particular, the second formant showed a distinct bimodal frequency distribution, suggesting a formant modulation (possibly involving the trunk) as found in savanna elephants [60,84]. The results also suggest an intentional modulation of the second formant, because its center frequency varied independently of variation in the fundamental frequency, which is usually related to motivational state [39].

### 3.4. Call Combination

Human languages construct meaningful expressions through the flexible combination of acoustic units into more complex utterances, generally referred to as syntax. Phonological syntax (or phonology) constructs words out of acoustic units that are not themselves meaningful. Lexical syntax generates *compositional* messages (i.e., sentences) to which each unit contributes its own meaning [12,87,88]. Two types of syntax, analogous to phonological and lexical syntax in human language, were recognized by Marler [87]. Phonological syntax is found among various species ranging from birds to gibbons and was traditionally viewed as the simpler and evolutionarily older form [89], however, others suggest that lexical syntax, even though more restricted to non-human primates, may have evolved first [90]. Call combinations have rarely been investigated in detail in terrestrial mammals other than primates (but see [91,92]), and here, most studies have focused on predator alarm calls (e.g., [93]). Given the inherently social nature of human language, studying call combinations produced in social contexts seems critical. Investigating the potential for lexical and phonological syntax in the combinatorial calls of the two African elephant species, at different levels of social complexity, may shed light on the evolutionary sequence producing the two forms of syntax in human language.

Both species of African elephants habitually combine their characteristic low-frequency rumble vocalizations with broadband calls (here loosely referred to as roars, Figure 1). Using forest elephant recordings conducted at two forest clearings using passive acoustic recordings, as well as recordings collected at several savanna elephant sites, Pardo et al. [43] compared the structure of these combinatorial calls between the two species. Forest elephants and savanna elephants appear to exhibit a similar repertoire of rumble-roar combinations, but forest elephants produced a higher proportion of combination calls than savanna elephants (forest elephants: 4–8% of calls, savanna elephant: 1–4%) and the proportion of different combination types differed significantly between the two species, the cause of which appears to be socio-ecological rather than phylogenetic [43]. In savanna elephants, combination calls were most often produced by immature individuals and least often by adult males and in diverse contexts, including agonistic, affiliative and sexual interactions, as well as related to nursing, separation and disturbance [43]. 

### 3.5. New Findings

**Figure 4:**
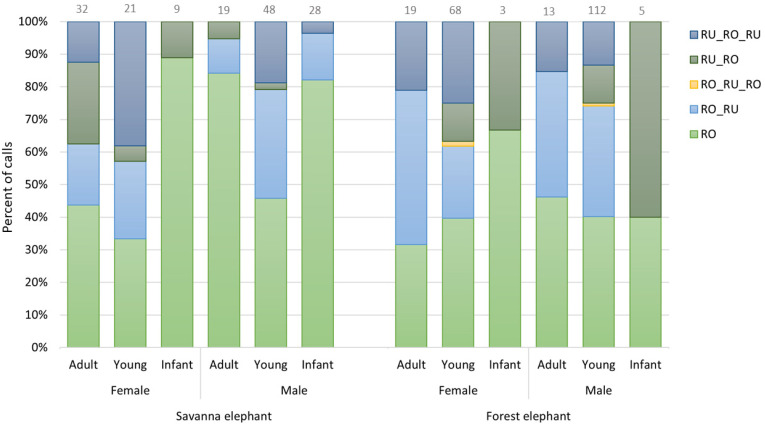
Percent proportion of roars and roar-rumble combinations produced by different age and sex classes of savanna and forest elephants. Numbers above each bar indicate the sample size for the respective age-sex class (savanna elephants: N = 157, forest elephants: N = 220).

Our new data on the use of combinatorial calls by forest elephants at Dzanga Bai and additional data on savanna elephants from Amboseli reveal that, in accordance with findings by Pardo et al. [43], the use of call combinations, as opposed to stand alone roars, appears to be more prevalent across age/sex classes in the forest as compared to the savanna elephant (combination calls made up 60% of forest elephant roars and only 43% of savanna elephant roars; Figure 4). 

**Figure 5:**
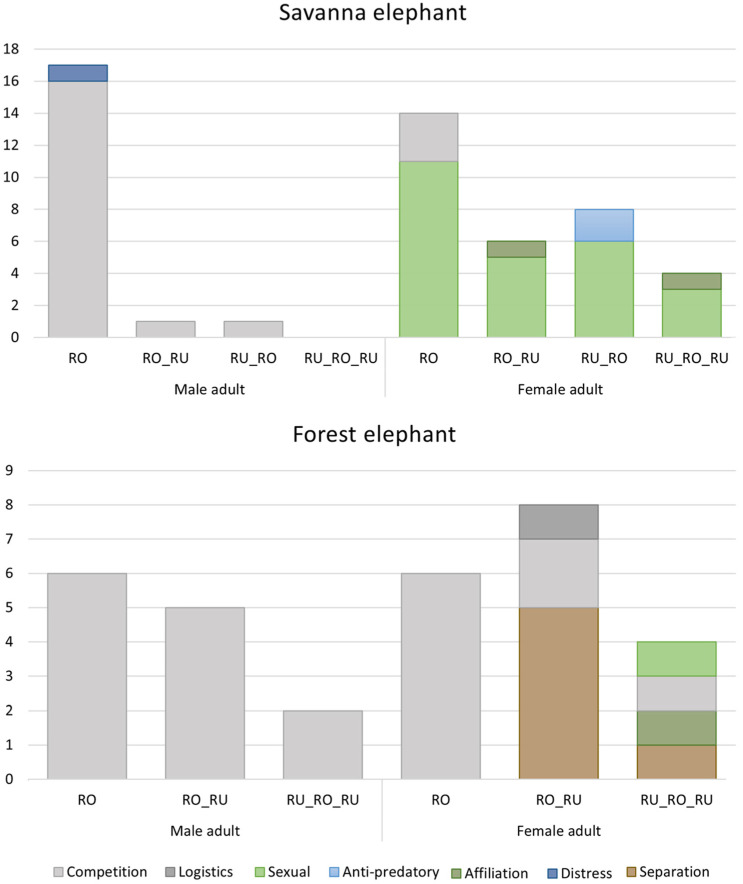
Number of roars used singularly, and roar-rumble combinations produced in different contexts by adult male and female savanna and forest elephants (savanna elephants: N = 51; forest elephants: N = 32).

Our first comparison of the contextual use of roars and combination calls produced by adults of the two species suggests that roars, as well as combination calls of adult male elephants of both species are produced predominantly in competition contexts; however, adult male forest elephants appear to produce many more combination calls than adult male savanna elephants (Figure 5). Adult females of both species appear to produce roars and combination calls in a broader range of contexts than do adult males and more combination calls than stand-alone roars. As expected for syntactic systems, in females of both species combining a roar with a rumble appeared to modify the contexts in which roars were being used. However, contextual use of roars and combinations differed between the two species. In female forest elephants, the combination with a rumble appeared to increase the number of contexts in which a roar was produced. While roars were observed only in competition contexts, combinations of rumbles and roars were associated with five contexts, including competition as well as sexual, affiliative, separation and logistical contexts. In Amboseli adult female savanna elephants, roars and combination calls were predominately observed in a sexual context (78%, N = 32), but a more detailed look at the specific behavior reveals that combination calls also appear to modify the context. The majority of roars or combination calls given in a sexual context were by an estrous female being chased by a male (N = 18). Of these, 11 were roars. The remaining calls given in a sexual context were all combination calls and occurred in more social settings, following a mating (mating-pandemonium) or when females were greeting a musth male (female-chorus). Likewise, combination calls were also given in affiliative contexts and when a family was mobbing predators, while the remaining stand-alone roars were observed in a competitive context (Figure 5). 

### 3.6. Environmental Constraints on Vocal Mediation of Interactions

Vocal mediation of social interactions may facilitate the formation of complex social organizations, particularly when individuals cannot engage directly [4]. Estimating the distance over which vocalizations can be detected and interpreted is key to our understanding of the extent of such vocal coordination, but, effective detection distances have been determined for only a few species [94,95,96,97,98]. Detection distances are limited as sound experiences reverberation and absorption during propagation, leading to the attenuation and distortion of the acoustic signal. The strength of such degrading effects depends on frequency-related and temporal features of the acoustic signal in relation to environmental characteristics. For example, in forest habitats with dense vegetation, high pitched signals attenuate faster than they would in open habitats, such as savannas [99]. 

Savanna elephants appear to be able to recognize each other from the harmonic structure of some rumbles up to a distance of 2.5 km, enabling them to coordinate interactions over long distances and to form extensive vocal recognition networks [24,84]. Simulation models suggest that when conditions are optimal, savanna elephants may be able to detect rumbles over distances of up to 10 km [100]. Models of the attenuation of forest elephant rumbles based on amplitude measurements of rumbles recorded in a Central African rainforest suggest that forest elephant rumbles attenuate faster than savanna elephant rumbles [98]. Under optimal conditions when ambient sound is lowest, forest elephants may be able to detect a rumble of average fundamental frequency and source pressure level up to 3.2 km. However, under average ambient conditions, an average rumble would be completely masked by background noise at only 800 m, with the harmonic structure of the majority of rumbles attenuating after only 100 m. Such short detection distances suggest that the ability to coordinate interactions over long distances is limited in forest elephants which may severely constrain the maintenance of social complexity compared to levels documented in savanna elephants. Hedwig et al.’s [98] estimation of detection distance assumed a noise perception of forest elephants similar to that of other vertebrates. However, detailed information on hearing sensitivity and auditory filters is lacking for any species of elephants, and for frequencies below 100 Hz for terrestrial vertebrates in general, but critically needed to better understand the functioning of low-frequency rumble vocalizations within the elephant social systems.

## 4. Discussion

The social complexity hypothesis for communication states that the range and frequency of social interactions drive the evolution of complex communication skills enabling individuals to flexibly convey a wide range of information to maintain their social relationships (e.g., [3]). Intriguingly, the first insights into the vocal communication system of the elusive forest elephant suggest that African elephants may contradict the social complexity hypothesis. The socially less complex forest elephant appears to exhibit communication skills that are at least as sophisticated as savanna elephants, a species with one of the most complex social systems observed among mammals. Comparison based solely on the acoustic structure of rumbles of both species indicates a seemingly larger but equally highly graded repertoire of rumble types in forest elephants as compared to savanna elephants [39,57]. In addition, both species appear to exhibit communication skills that may increase the generativity of their vocal repertoires: the modulation of formant structure through the intentional inclusion of the trunk into vocal production [39,60,84] and the combination of roars with rumbles into contextually more complex call combinations [43,101].

These preliminary findings suggest that the complex communication skills observed in savanna elephants may be ancestral traits that evolved in the context of a forest-dwelling last common ancestor, instead of constituting socio-cognitive adaptations to the environmental conditions of a savanna habitat. Our study modelling the attenuation of forest elephant rumbles suggests that sound transmission conditions in dense rainforests may inhibit long distance communication and, thus, vocal coordination of social relationships. As such, the potentially sophisticated communication skills of extant forest elephants, and likely those of their forest-dwelling ancestors, permit the transmission of exceptionally complex information, but only within the context of their small groups. Increased predation pressure by large pack-hunting mammals and seasonal abundance of food resources in the savanna may have driven the formation of larger social groups. The exceptionally low frequencies of rumble vocalizations and the power with which these sounds can be produced [37] are likely a byproduct of the elephants’ large body size. Powerful, very low frequency calls paired with reduced attenuation in the open savanna, may have allowed savanna elephants a larger spatial range over which information can be transmitted and interactions between groups mediated.

Our first insights into the patterns of the vocal complexity in *Loxodonta* appear to challenge the social complexity hypothesis and offer an exciting field for future comparative research. The emergence of complex communication skills may be driven by other factors than social complexity alone [3], none of which, however, appear to be accountable for the complex communication skills in forest elephants. First, the decreased visibility in the rainforest would predict a less graded rumble repertoire in the forest elephants compared to the savanna elephant to reduce ambiguity in signal interpretation, but this is not evident [39]. Second, predation pressure may bring about an increase in repertoire size through the evolution of predator specific alarm call types [102] or call combinations [103]. Despite humans currently and historically being key predators of both species of African elephants, savanna elephants are assumed to experience higher predation pressure due to the presence of large pack hunting predators, which are absent in the rainforest environment. Recent research documents that variation in the acoustic structure of savanna elephant rumbles encodes context specific information about the presence of external threats [61,85]. Whether forest elephant rumbles encode similar information currently remains unknown. Third, a high overlap of a species’ ecological or acoustic niche with other species may drive signal diversity to enhance species recognition [104]. However, such overlap should be minimal, as elephants occupy an acoustic niche reaching into the infrasound range, largely inaccessible to the vocal production of most other sympatric species. Lastly, communicative complexity may emerge through neutral evolutionary processes and constitute the result of mutation and recombination independently of any social or ecological factors [105]. Phylogenetic analysis can be used to test for neutral evolution as closely related species are expected to exhibit similar levels of complexity compared to evolutionarily more distant species. The elephantid family consists of only three extant species, including the Asian elephant *Elephas maximus*. Results regarding the repertoire of combinatorial calls in the three species, however, do not indicate that neutral evolutionary processes have played a role as all three elephant species exhibit a similar repertoire size of call combinations [43]. 

Factors that remain underexplored as potential drivers of vocal complexity are life history traits and associated levels of parental care and social learning. Among terrestrial mammals, the forest elephant displays an exceptionally slow and low reproductive rate with a late median age of primiparity of 23 years and long median interbirth intervals of 5.6 years [106]. While reproductive rates generally correlate negatively with body size [107], forest elephants exhibit substantially lower reproductive rates and slower life histories compared to savanna elephants, despite their smaller body size [106]. As suggested for the long slow life histories characteristic for primates [108], these low reproductive rates may be driven by challenges associated with foraging in a highly variable forest environment on food sources, such as fruit with seasonally limited availability and patchy distribution, or foliage often high in toxins [106]. Slow life histories go hand in hand with extended parental care and a potentially high degree of social learning. The complex communication system observed in forest elephants may reflect the strong bonds between female forest elephants and their offspring which facilitate social learning needed to maximize survival in a highly complex environment. Similarly, strong social bonds have been put forth to explain complex cognitive abilities in some monogamous pair-bonded bird species [109].

In our first comparison, the frequency of rumbling in different contexts showed a marked discrepancy between the two species of *Loxodonta*. Here, we speculate whether our observations represent a species- or rather site-specific distinction, shaped by environmental features particular to the sites. Observations of forest elephants took place at Dzanga Bai, a social arena in a dense forest where elephants gather in large numbers due to the distinct availability of minerals through monopolizable pits, whereas observations of savanna elephants took place largely in Amboseli’s open grasslands, with its saline soils and numerous swamps. These site-specific differences likely account for the large discrepancy between the frequency of rumbles given in a competitive context as competitive interactions constitute the most frequently observed social interaction between forest elephants at Dzanga Bai (unpublished data DH). Another large difference occurs in the context of separation. In both species, calves were responsible for the majority of calling in a separation context. It is possible that forest elephant calves spend more time further from their mothers or siblings than do savanna elephants because their survival is not threatened by large pack-hunting predators. While savanna elephant calves also habitually wander further from their mothers to socialize, Dzanga Baimay provide forest elephant calves with a perhaps unusual opportunity for social interaction and exploration. However, the large differences between the frequency of rumbles given in affiliative, logistical, and even sexual, nursing and anti-predatory contexts point more to species-specific differences. Given that savanna elephants have larger families and more frequent fission-fusion events [21,22,23], we would expect to see more frequent greetings and bonding events, and more calling related to the coordination of members’ movements and their location relative to one another in savanna relative to forest elephants. Yet, since forest clearings are assumed to constitute important social arenas [35], the degree of difference we observed between the two species is surprising. In addition, forest elephant females exhibit an older age of first reproduction compared to savanna elephant females (forest elephant: 23 years [106], savanna elephant: 14 years [110]), along with longer interbirth intervals (forest elephant: 5.6 years [106], savanna elephant:~4.5 years [110]) and generation lengths (forest elephant: 31 years, savanna elephant: 23 years [111]). As such, estrous behavior, mating and even nursing and weaning events would be expected to occur less frequently in forest elephants than among savanna elephants. Furthermore, the difference between the frequency of rumbles that occurred during anti-predatory events can likely be explained by the lack of large predators in the rain forest. In summary, we suggest that differences between the two species in the degree of fission-fusion sociality and life history parameters may account for more of the observed discrepancies in the frequency of calling by context than do site-specific differences.

Conclusions are drawn regarding differences in vocal complexity exhibited by the two species of African elephants, however, they suffer from insufficient information on forest elephants and a lack of directly comparable data and methods for both species. As we are only beginning to understand forest elephant behavior, any conclusions regarding the level of vocal complexity in this species must be drawn with caution. Yet, the first insights presented here can serve as hypotheses guiding much needed future studies on the structure and contextual use of vocalizations of these elusive animals. Direct observations of forest elephants are difficult. The dense vegetation in the Central African rainforest poses extreme limitations in visibility and it is not possible to observe them from vehicles as is habitually done in savanna elephant studies. Forest clearings, however, such as Dzanga Bai where elephants venture out in the open and aggregate in large numbers, provide unique opportunities to observe forest elephant behavior from observation platforms [32]. Our results show that the Bai studies can provide comprehensive insights into the contextual use and structure of the vocal repertoire of forest elephants. However, our results are based on calls that we were able to hear and localize with sufficient confidence to define context, and, as such, low amplitude rumbles and those produced in subtle contexts currently remain unexplored. The development of advanced acoustic recording methods, such as localization of calls using acoustic arrays consisting of time-synchronized recording units, paired with simultaneous high resolution video recording will be imperative to advance our understanding of forest elephant rumbles. Not surprisingly, our work also demonstrates that not all aspects of forest elephant vocal communication can be observed at forest clearings. Bai aggregations represent highly social and unique contexts, where forest elephants spend only a fraction of time. A better understanding of forest elephant social and vocal communication critically relies on the development of innovative methods to study them in their forest environment. Passive acoustic recording in combination with camera traps as well as acoustic recorders on radio collars are promising, yet challenging, methodological avenues that require further development.

If comprehensive studies of forest elephants are a challenge, so are conducting meaningful comparisons with savanna elephants. The recently released The Elephant Ethogram, an extensive repository of savanna elephant behavior [41], provides an excellent reference point for forest elephant observational studies and the groundwork for future comparative work on the contextual use of rumbles in both species. Our comparison of the contextual use of rumbles and rumble-roar combinations in the two species, however, highlights how site- or population-specific features could lead to erroneous conclusions regarding species-level differences. In addition, despite the considerable number of insightful studies dedicated to savanna elephants, various aspects regarding their vocal communication require more research. Conclusions drawn about the importance of rumble vocalizations in the maintenance of relationships in a fission-fusion setting are largely based on playback experiments simulating optimal sound transmission conditions [24,84,112] and focused on specific rumble types, such as contact and estrous rumbles. Attenuation models or playback experiments considering the natural range of conditions under which vocal communication may take place in savanna elephants are needed to get a more realistic view of the role of long-distance communication in facilitating their complex social organization [98].

## 5. Conclusions

First insights into the vocal communication system of the elusive forest elephant suggest complex communication skills. These findings contradict the social complexity hypothesis, which predicts a less complex communication system in the forest elephant compared to the socially highly complex savanna elephants. We propose the exceptionally slow and low reproductive rate and associated high levels of parental care in forest elephants as drivers of vocal complexity in this species. However, conclusions regarding differences in vocal complexity between the two species need to be drawn cautiously. A better understanding of vocal complexity in the genus *Loxodonta* will depend on continuing advancements in remote data collection technologies, such as passive acoustic recording and camera trapping, to overcome the challenges of observing forest elephants in their dense rainforest habitat. Finally, inconsistent methodologies currently strongly inhibit direct comparison between forest and savanna elephant vocal behavior. Studies based on directly comparable data and methods, quantifying both structural and contextual variability in the production of rumbles in both species are critically needed to understand patterns of vocal complexity within the genus *Loxodonta*, and beyond.

## Figures and Tables

**Table 1:** Behavioural contexts of rumbles, roars and rumble-roar combinations produced by forest elephants at Dzanga Bai, and corresponding contexts for savanna elephants, as defined in The Elephant Ethogram [41] *. To make this direct comparison, we have aligned contexts defined in The Elephant Ethogram to match the broader contexts described for forest elephants. The rumble sub-types, roars and combination calls listed in parentheses are those included in our preliminary analyses in Figure 2, Figure 3, Figure 4 and Figure 5 and do not represent all rumbles (e.g., husky-cries or “grunts” by new-borns, baroo-rumbles) or call-types (e.g., trumpets, nasal trumpets, snorts) given in these contexts. To attempt the best fit with forest elephant contexts, savanna elephant calls categorized under the context Protest and Distress have been divided into Protest versus Distress, and those categorized under Calf Reassurance and Protection have been separated into those that occurred in an affiliative versus separation context.

Forest Elephants		Savanna Elephants	
Context	Description	Context	Definition
Affiliation	Individuals in the Bai, or upon entering the Bai approaching one another, or walking up to a stationary individual, usually leading to benign physical contact. Individuals may stay in proximity, feeding, traveling, resting, or sharing a mineral pit.	Affiliative	Vocalizations given during the formation and maintenance of social bonds with other elephants (greeting-rumble, little-greeting-rumble, rumbles associated with greeting-ceremonies, some bonding-ceremonies and reconciliations, rumble-roar, rumble-roar-rumble).
		Birth	Vocalizations given by a newborn, its family members, or nearby elephants during, or within 24 h prior to, or after, parturition, that relates to the birth (coo-rumble, bonding-rumble).
		Calf Reassurance and Protection	Vocalizations given when defending, protecting, helping, guiding, comforting or consoling calves, or by calves responding to such actions (coo-rumble, as-touched-rumble).
Competition	Conflict over access to resources, mostly mineral pits, occasionally grass. Includes displacements, avoidance interactions, nonphysical and physical aggression, as well as disputes by individuals sharing a mineral pit.	Aggressive	Vocalizations given when displacing, intimidating, threatening or attacking other elephants (rumble).
		Submissive	Vocalizations given in submission to avoid aggression and/or injury by other elephants (V8-rumble).
		Protest	Vocalizations given when in pain, or when complaining or protesting at some perceived wrong—*in a competitive situation* (grumble-rumble, roar, roar-rumble, rumble-roar).
Separation	Individuals travelling alone at a large distance from associates or when associates have left the clearing. Individuals often wander around aimlessly, smelling ground with their trunk, frequently stopping to listen, or running in distress with ears and tail erect.	Separation	Vocalizations given by a calf or juvenile elephant when separated from its mother or family. May call repeatedly while wandering around sniffing the ground or air with the trunk, stopping to listen, or running in distress with ears spread and tail raised (separated-rumble, roar-rumble, rumble-roar-rumble).
Anti-predatory	Individual mobbing (charging, kicking dust or flinging trunk) animal of other species, or when in a state of alert (ears erect and tail up) upon visitors arriving at the observation platform.	Vigilance	Vocalizations given when attending to, or alerting companions to, possible danger posed by potential predators (comment-rumble).
		Conflict and Confrontation	Vocalizations given when signaling hostility toward a perceived non-elephant threat (rumble).
		Coalition Building	Vocalizations given during the formation of a defensive coalition (rumble).
		Mobbing and Attacking	Vocalizations given by an elephant, or a group of elephants acting cooperatively, when advancing upon, harassing, or attacking a non-elephant threat (rumble, rumble-roar).
		Avoidance	Vocalizations given when evading actual or perceived dangers posed by non-elephant threats (rumble).
Sexual	Female presenting her hindquarters allowing males to inspect genitals, often urinating while doing so. Males contesting over females. Females, often in distress, avoiding a male who is pursuing them.	Advertisement and Attraction	Vocalizations given by adult male and adult female elephants in the advertisement of sexual state and the attraction of, and search for, mates (musth-rumble, rumbles given during a female-chorus, estrous-running-rumble, estrous-roar, estrous-running-rumble-roar, roar, roar-rumble, rumble-roar, rumble-roar-rumble).
		Courtship	Vocalizations given by adult male and female elephants during consorting and mating (estrous-running-rumble, estrous-roar, estrous-running-rumble-roar, estrous-rumble, calls within a mating-pandemonium or female-chorus including, rumbles, roar-rumble, rumble-roar, rumble-roar-rumble).
Nursing	Calf approaching or walking parallel along mother’s side with trunk raised, often touching mother’s side, leg or breast. The mother usually stops and starts nursing.	Calf Nourishment and Weaning	Vocalizations given by calves when attempting to meet their nutritional requirements and during the process of weaning (begging-rumble, cry, cry-rumble, roar, roar-rumble, rumble-roar, rumble-roar-rumble).
Logistics	Concerted group movement and group coordination as individuals travel or leave the clearing together.	Movement, Space and Leadership	Vocalizations given when maintaining spatial proximity, initiating group movement or influencing its timing, direction or form (let’s-go-rumble, cadenced-rumble, contact-rumble).
		Distress	Vocalizations given when in pain, or when complaining or protesting at some perceived wrong *in a non-competitive situation* (roar, roar-rumble, rumble-roar-rumble).
Unspecific	Individuals are stationary feeding on grass, accessing mineral pits or resting, often within 20 m of associates, no obvious social interaction or change in activity, often exchanging rumbles.		

* Savanna elephant rumble and combination calls used in the comparative analysis are given in parentheses. Postures, gestures and behaviours associated with these contexts and calls can be found in the Ethogram Table and via the Search Portal in The Elephant Ethogram.

## Data Availability

Data will be made available upon request.

## Supplementary Materials

The following are available online at https://www.mdpi.com/article/10.3390/ani11113071/s1, Table S1: Number of forest elephant rumbles by context, as well as sex and age of the caller, Table S2: Number of savanna elephant rumbles by context, as well as sex and age of the caller, Table S3: Contextual differences in the duration of rumbles produced by forest elephants, controlled for age of caller, Table S4: Contextual differences in the duration of rumbles produced by savanna elephants, controlled for age caller.
