# Supplementary material for: Does Social Complexity Drive Vocal Complexity? Insights from the Two African Elephant Species
Source: Animals (Basel). 2021 Oct 27;11(11):3071. doi: 10.3390/ani11113071 (PMC8614502; doi:10.3390/ani11113071)
Supplement: Supplementary file 1 [file animals-11-03071-s001.zip › animals-1383271-supplementary.pdf]

Electronic supplementary material 1. Sample sizes and detailed analysis results

Title: Does social complexity drive vocal complexity – insights from the two African elephant species

Daniela Hedwig[1a], Joyce Poole[2] and Petter Granli[2]

[1] Elephant Listening Project, K. Lisa Yang Center for Conservation Bioacoustics, Cornell Lab of Ornithology, Cornell University, Ithaca, USA

[2] ElephantVoices, Sandefjord, Norway

[a] corresponding author; email: dh646@cornell.edu

Table S1. Number of forest elephant rumbles by context, as well as sex and age of the caller. A total of N=304 rumbles were used to investigate the overall contextual use of rumbles in forest elephants (figure 3 in main text). A subset of N=246 rumbles was used to investigate contextual differences in the acoustic structure of rumbles (while controlling for age of the caller). This subset excluded rumbles produced by individuals of unknown age and sex, and due to small sample sizes, those produced in “unspecific”, “sexual” and “antipredatory” context, as well as those produced by infants of both sexes and adult males. Rumbles excluded from the subset are presented in italics. The sample sizes per context of the large dataset are presented in the total column with the sample sizes for the data subset in parentheses. Age groups: infant (“inf”): <1 year; juvenile (“juv”):>1 to <6 years; subadult (“sa”): >6 to 20 years; adult (“ad”): >20 years.

	**Females**					**Males**					**Unknown sex**					**Total**
	**inf**	**juv**	**sa**	**ad**	**un**	**inf**	**juv**	**sa**	**ad**	**un**	**inf**	**juv**	**sa**	**ad**	**un**	
**Affiliation**	*1*	1	11	25	0	0	1	8	0	0	0	*1*	0	0	*2*	50 (46)
**Competition**	0	7	7	8	0	0	21	15	*1*	2	0	0	0	0	*4*	65 (60)
**Antipredatory**	0	0	0	1	0	0	0	0	0	0	0	0	0	0	*1*	2 (0)
**Logistics**	0	0	6	7	0	0	0	0	0	0	0	0	0	0	0	13 (13)
**Nursing**	0	7	0	0	0	*1*	3	2	0	0	0	*3*	0	0	*4*	20 (12)
**Separation**	*1*	8	45	27	*6*	*2*	17	18	0	0	*8*	*10*	0	0	*1*	143 (115)
**Sexual**	0	0	0	*1*	0	0	0	0	*4*	0	0	0	0	0	*2*	7 (0)
**Unspecific**	0	0	0	*4*	0	0	0	0	0	0	0	0	0	0	0	4 (0)
																304 (246)

Table S2. Number of savanna elephant rumbles by context, as well as sex and age of the caller. A total of N=3006 rumbles were used to investigate the overall contextual use of rumbles in savanna elephants (figure 3 in main text). A subset of N=1177 rumbles for which acoustic measurements were available was used to investigate contextual differences in the acoustic structure of rumbles (while controlling for age of the caller). This subset excluded rumbles produced by individuals of unknown age. The sample sizes per context of the large dataset are presented in the total column with the sample sizes for the data subset in parentheses. "Mixed sex&age" as well as "Mixed female age" refer to rumbles produced by several individuals of different sex and ages calling simultaneously.

	**Male**			**Female**				**Unknown sex**				**Mixed sex&age**	**Mixed female age**	**Total**
	**<5**	**5<10**	**>10**	**<5**	**5<10**	**>10**	**un**	**<5**	**5<10**	**>10**	**un**			
**Affiliative**	47	10	4	45	126	681	30	14	0	0	22	6	15	1000 (426)
**Competition**	37	8	11	10	10	15	0	10	1	1	1	0	0	104 (55)
**Anti-predatory**	0	0	0	1	0	76	1	1	0	0	8	0	1	88 (11)
**Logistics**	10	13	3	13	34	747	54	5	0	0	70	0	0	949 (349)
**Nursing**	168	5	0	68	1	0	0	46	1		24	0	0	313 (136)
**Separation**	63	2	0	69	0	1	0	66	0	0	0	0	0	201 (89)
**Sexual**	0	0	138	1	6	170	32	1	0	0	2	1	0	351(111)
														3006 (1177)

Table S3. Contextual differences in the duration of rumbles produced by forest elephants, controlled for age of caller. Output of linear regression model with duration as response and the context categories and age class of caller as predictor variables. N=246. Due to small sample size for these age classes rumbles produced by infants and adult males were not included in this analysis.

	Estimate	SE	P-value
Intercept	3.452	0.221	<0.001
Adult vs			
Subadult	-1.530	0.184	<0.001
Juvenile	-1.987	0.229	<0.001
Competition vs.			
Affiliation	0.896	0.247	<0.001
Separation	1.450	0.192	<0.001
Nurse	0.118	0.370	0.749
Logistics	1.578	0.367	<0.001
Affiliation vs.			
Separation	0.553	0.209	0.009
Nurse	-0.778	0.405	0.056
Logistics	0.682	0.361	0.062
Separation vs.			
Nurse	-1.331	0.367	<0.001
Logistics	0.129	0.342	0.707
Logistics vs.			
Nurse	-1.460	0.489	0.003

Table S4. Contextual differences in the duration of rumbles produced by savanna elephants, controlled for age caller. Output of linear regression model with duration as response and the context categories and age of caller (in years) as predictor variables. N=1177.

	Estimate	SE	P-value
Intercept	2.438	0.459	<0.001
Age	0.033	0.003	<0.001
Competition vs			
Affiliation	0.759	0.215	<0.001
Separation	0.025	0.253	0.922
Nurse	-0.602	0.236	0.011
Logistics	1.858	0.224	<0.001
Sexual	1.204	0.258	<0.001
Anti-predatory	-0.055	0.496	0.911
Affiliation vs			
Separation	-0.734	0.180	<0.001
Nurse	-1.361	0.156	<0.001
Logistics	1.099	0.110	<0.001
Sexual	0.445	0.164	0.007
Anti-predatory	-0.814	0.453	0.073
Separation vs			
Nurse	-0.627	0.201	0.002
Logistics	1.833	0.194	<0.001
Sexual	1.179	0.233	<0.001
Anti-predatory	-0.080	0.483	0.869
Logistics vs			
Nurse	-2.460	0.173	<0.001
Sexual	-0.654	0.162	<0.001
Anti-predatory	-1.913	0.452	<0.001
Sexual vs			
Anti-predatory	-1.259	0.466	0.007
